# The Family Relation

**Published:** 1882-05

**Authors:** 


### THE FAMILY RELATION.

"He was always allowed to have his own way," was the record a few days ago, of a well-dressed young man who was found dead in the road, with a bullet hole through the back of his head. He was the only son of a wealthy widow, who furnished him with all the money he wanted, so that he might enjoy himself; with the result that he fell into bad company, and dissipated habits and evil associations, and was murdered on the distant shores of California for the jewelry which he had on his person; leaving his mother in all her lonliness in her New England home, to pass the remainder of her life in bitter remorse for having acted on the mistaken principle, that the best way to have a child enjoy himself, was to allow him "to have his own way." Within a day or two, a murderer who had some education, and had enjoyed the advantages of good society, made the deliberate statement under the gallows:

"And now I want to say to all these people to respect their parents. When we throw all parental authority aside we commence a course of sin. It is terrible to be disobedient to parents Sabbath breaking and lying, too, are awful things. There are many here who have children, I want to state to them that it is their great duty to God and their children to guard them well in their youth, and give them good influences to surround them; to take them more into their confidence, and make home inviting and happy to them, and talk to them more. My father was a church member, and so was my mother; but they never gave me any advice. They went to their church every Sunday, but they left their religion at the church. They never explained to us the doctrines of the Bible. All parents should see that their children have a love for God, and they should let them know what the bible is. A great many children are running around in the streets, and they get them into the jails and that only makes them worse. We should make home happy, and a blessing for them. It is a duty that God devolves upon us. I hope God may pardon all our sins, and that he may receive my soul."

Such facts show that it is the houshold education which moulds the character, and that if the family relation was properly understood and its duties and obligations were properly met, a vast amount of crime, and destitution, and wretchedness, and premature death would be prevented.

. . . . . .

### FROST WORK.

There is beauty in a splendid education, where the mind has been trained in all the accuracy of mathematics, in all the elevating elegances of poetry, and painting, and sculpture, and music. And grammar, and logic, and rhetoric may have been thoroughly mastered and reduced to the practice of an accomplished writer and a finished orator, an abstract morality may pervade every line written, every uttered sentiment. But who does not know, that these things alone, never make a loveable character, there is beauty in it, as cold as the icicle, as dead as the marble, as transient as the clouds of morning, the body is without warmth, the heart without sympathy, the whole nature without love, beyond the circle of its own cold and dead, and magnificent self.

The son of a London Alderman, was William Beckford, left fatherless at ten, with an annual income of half a million of dollars. No pains were spared to give him a refined education. The great Mozart taught him music. Sir William Chambers gave him lessons in architecture. At the moment of his majority, he launched out upon the world, proud, haughty and accomplished, withdrew from men, encircled his domains with a two mile wall, razeed his father's residence, which had been built at an expense of more than a million of dollars, and erected another on its site, in mid-winter, men working day and night, and amid gorgeous palaces, in more than eastern magnificence, he passed his time in luxurious ease. He feasted in the contemplation of his own grandeur, but the day was not long enough for that, for at times when his whole establishment was lighted up with torches at mid-night, he would repair to a distant elevation, and gaze upon it by the hour. All this was the result, the very natural result of an "accomplished education." Had he been brought up in another school which would have invited out the affections, which would have trained him in the exercise of a benevolent nature; had he been been taught that the best way to happyfy himself was to be employed in happyfying others, he would most probably have lived to high purposes, and would have gone down to the grave with the benedictions of multitudes on his head, to be repeated as to his memory, by every successive generation, for all time. Instead of which his towers fell with their own weight, property depreciated, law suits terminated adversely, the sheriff entered where royalty had been refused, and he fell unpitied. Font Hill Abbey, like its owner, was levelled to the earth, and the wealth and the name of the author of *Vathek*, have faded away "like frost work before the sun." From this narration, many of the families of this land may derive instruction. The great aim of multitudes of parents, especially in cities, is to afford their daughters a splendid education, and the music of harps and guitars and pianos, is assiduously cultivated for months and years, instead of the infinitely more purifying and harmonizing "psalms and hymns, and spiritual songs" of Isaac Watts. The obscene and impossible mythology of classical poets is studied more than the real truths of scripture history, and the dance, and the ball, and the opera, are patronized before the prayer meeting, the lecture, and the sabbath worship. The result of all this is, our daughters grow up dressy, idle, proud, vain and worthless as to the real and necessary duties of life. They live in sham and show, and a vain parade, of which they soon get weary, because of their unsatisfying nature; life has no absorbing object; that of it which they see is tame and tasteless, there is nothing in it to stimulate them to energizing activities of mind or heart or body, and they pine away in listlessness and early disease, or, to protract for a brief space the flickering lamp of life, they feed it with opiates, or fill it with alcohol.

